# Suppression of F1 Male-Specific Lethality in *Caenorhabditis* Hybrids by *cbr-him-8*

**DOI:** 10.1534/g3.115.025320

**Published:** 2015-12-30

**Authors:** Vaishnavi Ragavapuram, Emily Elaine Hill, Scott Everet Baird

**Affiliations:** * Department of Biological Sciences, Wright State University, Dayton, Ohio 45435

**Keywords:** hybrid lethality, hybrid sterility, reproductive isolation, Haldane’s rule, Darwin’s corollary

## Abstract

Haldane’s Rule and Darwin’s Corollary to Haldane’s Rule are the observations that heterogametic F1 hybrids are frequently less fit than their homogametic siblings, and that asymmetric results are often obtained from reciprocal hybrid crosses. In *Caenorhabditis*, Haldane’s Rule and Darwin’s Corollary have been observed in several hybrid crosses, including crosses of *Caenorhabditis briggsae* and *C. nigoni*. Fertile F1 females are obtained from reciprocal crosses. However, F1 males obtained from *C. nigoni* mothers are sterile and F1 males obtained from *C. briggsae* die during embryogenesis. We have identified *cbr-him-8* as a recessive maternal-effect suppressor of F1 hybrid male-specific lethality in this combination of species. This result implicates epigenetic meiotic silencing in the suppression of F1 male-specific lethality. It is also shown that F1 males bearing a *C. briggsae* X chromosome are fertile. When crossed to *C. briggsae* hermaphrodites or F1 females derived from *C. briggsae* hermaphrodites, viable F2 and backcross (B2) progeny were obtained. Sibling males that possessed a *C. nigoni* X chromosome were sterile. Therefore, the sterility of F1 males bearing a *C. nigoni* X chromosome must result from dysgenic interactions between the X chromosome of *C. nigoni* and the autosomes of *C. briggsae*. The fertility of F1 males bearing a *C. briggsae* X chromosome provides an opportunity to identify *C. nigoni* loci that prevent spermatogenesis, and hence hermaphroditic reproduction, in diplo-X hybrids.

Reproductive isolation refers collectively to all genetic mechanisms that prevent or limit gene flow between populations (Mayr 1963; Coyne and Orr 2004). These mechanisms can be divided into two discrete categories, prezygotic mechanisms that prevent mating or fertilization and postzygotic mechanisms that decrease the fitness hybrid progeny. Most genetic models of reproductive isolation invoke dysgenic interactions among two or more loci (Dobzhansky 1936; Muller 1940, 1942; Wu 2001; Lindtke and Buerkle 2015). Within populations, interactions among these genes are maintained. Between populations, interactions among these genes are disrupted. Genes involved in reproductive isolation “are ordinary genes that have normal functions within species” (Orr *et al.* 2004).

Postzygotic mechanisms of reproductive isolation include hybrid sterility and hybrid lethality. Genes involved in hybrid sterility and inviability include a receptor tyrosine kinase, transcription factors, nuclear pore proteins, and a histone H3 methyltransferase (Wittbrodt *et al.* 1989; Ting *et al.* 1998; Presgraves *et al.* 2003; Barbash *et al.* 2004; Tang and Presgraves 2009; Phadnis and Orr 2009; Mihola *et al.* 2009). While reproductive isolation may evolve through nonselective mechanisms (Mayr 1963), there is evidence that many of these and other ‘speciation genes’ are or have been under positive selection (Johnson 2010; Ting *et al.* 1998; Presgraves *et al.* 2003; Barbash *et al.* 2004; Tang and Presgraves 2009; Araripe *et al.* 2010; Hart *et al.* 2014). Therefore, speciation can result from adaptive evolution of normal cellular processes.

Two patterns frequently observed in postzygotic reproductive isolation are Haldane’s rule and Darwin’s corollary to Haldane’s rule. Haldane’s rule is the observation that when gender-specific differences are observed in hybrid fitness, it is generally the homogametic gender that is more fit (Haldane 1922; Laurie 1997; Coyne and Orr 2004). Darwin’s corollary to Haldane’s rule is the observation that reciprocal hybrid crosses often produce different results (Turelli and Moyle 2007). These patterns are of interest because of how they inform our understanding of speciation (Coyne and Orr 2004).

The primary explanation for Haldane’s rule is the dominance model (Wu and Davis 1993; Turelli and Orr 2000). The dominance model posits that most hybrid incompatibility genes are recessive. F1 female hybrids that are heterozygous for an X-linked hybrid incompatibility gene are viable. F1 male hybrids that are hemizygous for that gene are inviable. Support for this model in regard to hybrid lethality is especially strong (Wu and Davis 1993). The primary explanation for Darwin’s corollary is that F1 hybrids from reciprocal crosses have different mitochondria, different maternal contributions and F1 males have different X chromosomes (Turelli and Moyle 2007).

In the nematode genus *Caenorhabditis*, many species pairs are isolated by hybrid sterility and/or by hybrid lethality (Baird *et al.* 1992; Baird and Yen 2000; Woodruff *et al.* 2010; Kiontke *et al.* 2011; Kozlowska *et al.* 2011; Dey *et al.* 2012; Baird and Seibert 2013; Félix *et al.* 2014; Dey *et al.* 2014). Among these is the combination of *Caenorhabditis briggsae* and *Caenorhabditis nigoni* (Woodruff *et al.* 2010; Kozlowska *et al.* 2011). From crosses of *C. briggsae* males to *C. nigoni* females, fertile F1 adult females and sterile F1 adult males were obtained. Fertile adult females also are obtained from the reciprocal cross but all male hybrids die during embryogenesis. Therefore, both Haldane’s rule and Darwin’s corollary to Haldane’s rule are observed in crosses between *C. briggsae* and *C. nigoni*.

In this article, *cbr-him-8* is identified as a maternal-effect suppressor of F1 male-specific lethality in crosses of *C. nigoni* males to *C. briggsae* hermaphrodites. It also is demonstrated that F1 males derived from *cbr-him-8* mutant mothers that possess a *C. briggsae* X chromosome are fertile. Finally, it is shown that fertile adult progeny can be obtained from crosses of these *C. briggsae*-X-bearing F1 males to *C. briggsae* hermaphrodites or to F1 females derived from *C. briggsae* mothers.

## Materials and Methods

### Nematode strains and strain maintenance

**Figure 1:**
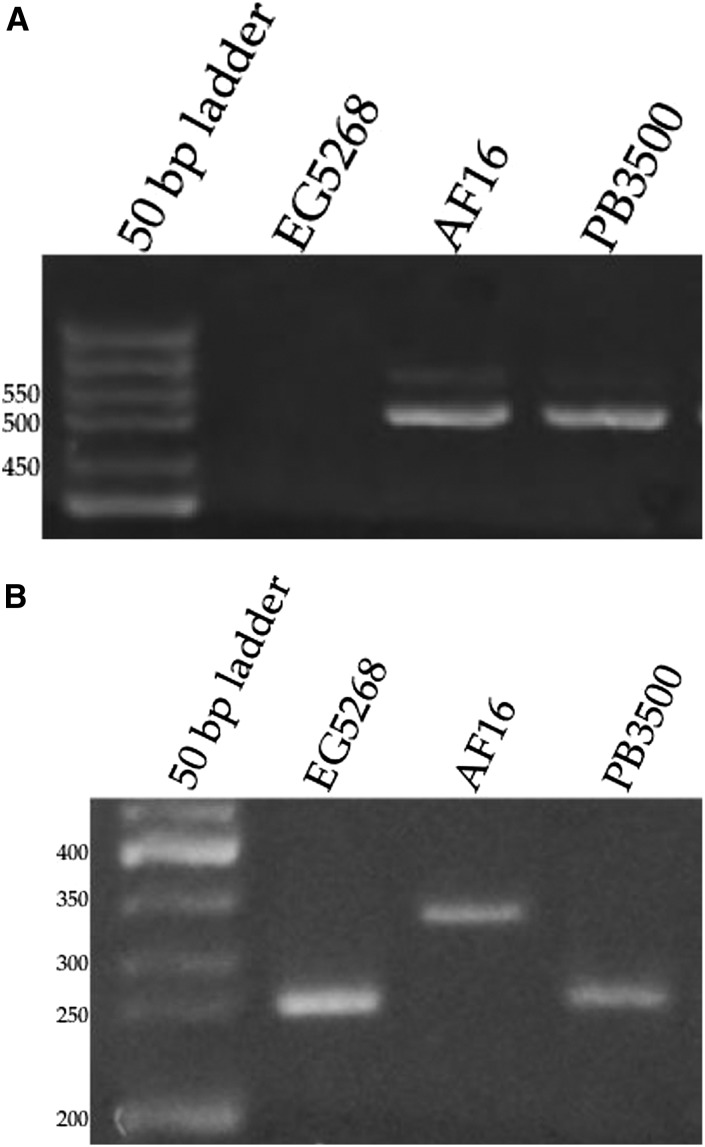
Confirmation of PB3500 cybrid genotypes. (A) Mitochondrial amplification products. Primers: *cbr-nad-5* - AGCCAAACTCTAACACCACCT and *cbr-nad-3* - TTCTTGGGGATTTTAGTTTCTGA. A 506 bp amplification product was expected from *C. briggsae* AF16 mitochondria. No product expected from *C. nigoni* EG5268 mitochondria. (B) Amplification products from the X-linked *cbr-vab-3* and *cni-vab-3* orthologs. Amplification products of 334 and 297 bp were expected from *C. briggsae* AF16 and *C. nigoni* EG5268, respectively. Primers: exon 4 - TGCACTCGGGCATACTGTAA and exon 6 - TGTACAACGGGCTCAGTCAG.

*C. nigoni* EG5268 (Kiontke *et al.* 2011; Félix *et al.* 2014) was provided by Marie-Anne Félix. *C. briggsae* AF16 (Fodor *et al.* 1983) was obtained from the *Caenorhabditis* Genetics Center. The *C. briggsae* AF16 derivatives RE980 [*cbr-him-8(v188) I*] (Wei *et al.* 2013) and RW20120 [*stIs20120 (pmyo2*::*GFP) X*] (Yan *et al.* 2012) were provided by Ron Ellis and Zhongying Zhao, respectively. PB192 [*cbr-him-8(v188) I*; *stIs20120 X*] was constructed from crosses of RE980 to RW20120. PB3500 was constructed from crosses of EG5268 males to AF16 hermaphrodites. Female progeny from this cross were backcrossed to EG5268 males for ten generations. Consistent with fixation of the *C. nigoni* nuclear genome, PB3500 had a female reproductive mode. Fixation of the *C. briggsae* AF16 mitotype and of the *C. nigoni* X chromosome in PB3500 was confirmed by amplification of species-specific mitochondrial and X chromosomal DNA products (Figure 1). Nematode strains were grown at 20° on lawns of *Escherichia coli* strain DA837. All strains used in this study are available from the *Caenorhabditis* Genetics Center.

### Crosses

Crosses always were of five males mated to three females or sperm-depleted hermaphrodites, and were conducted on freshly seeded mating plates (plates seeded with an approximately 1 cm spot of *E. coli*). Hermaphrodites were sperm-depleted by daily transfers for 4–5 d to fresh plates until egg laying ceased.

### Microscopy

Crosses and routine microscopy were conducted using stereomicroscopes at magnifications of 25–50×. Pharyngeal GFP fluorescence was scored using an M2Bio fluorescence microscope (Kramer Scientific). Analyses of gonadal morphology were conducted using DIC optics at a magnification of 400× on a Zeiss Axiovert 35M microscope.

### Reagents

All strains used in this study are available from the *Caenorhabditis* Genetics Center.

### Data availability

Supplemental data on control crosses between *C. nigoni* EG5268 males and *C. briggsae* RW20120 hermaphrodites is available at figshare.com/articles/EG5268_x_RW20120_suppl_data_xlsx/2058864.

## Results

### F1 male-specific lethality is suppressed by cbr-him-8(v188)

**Figure 2:**
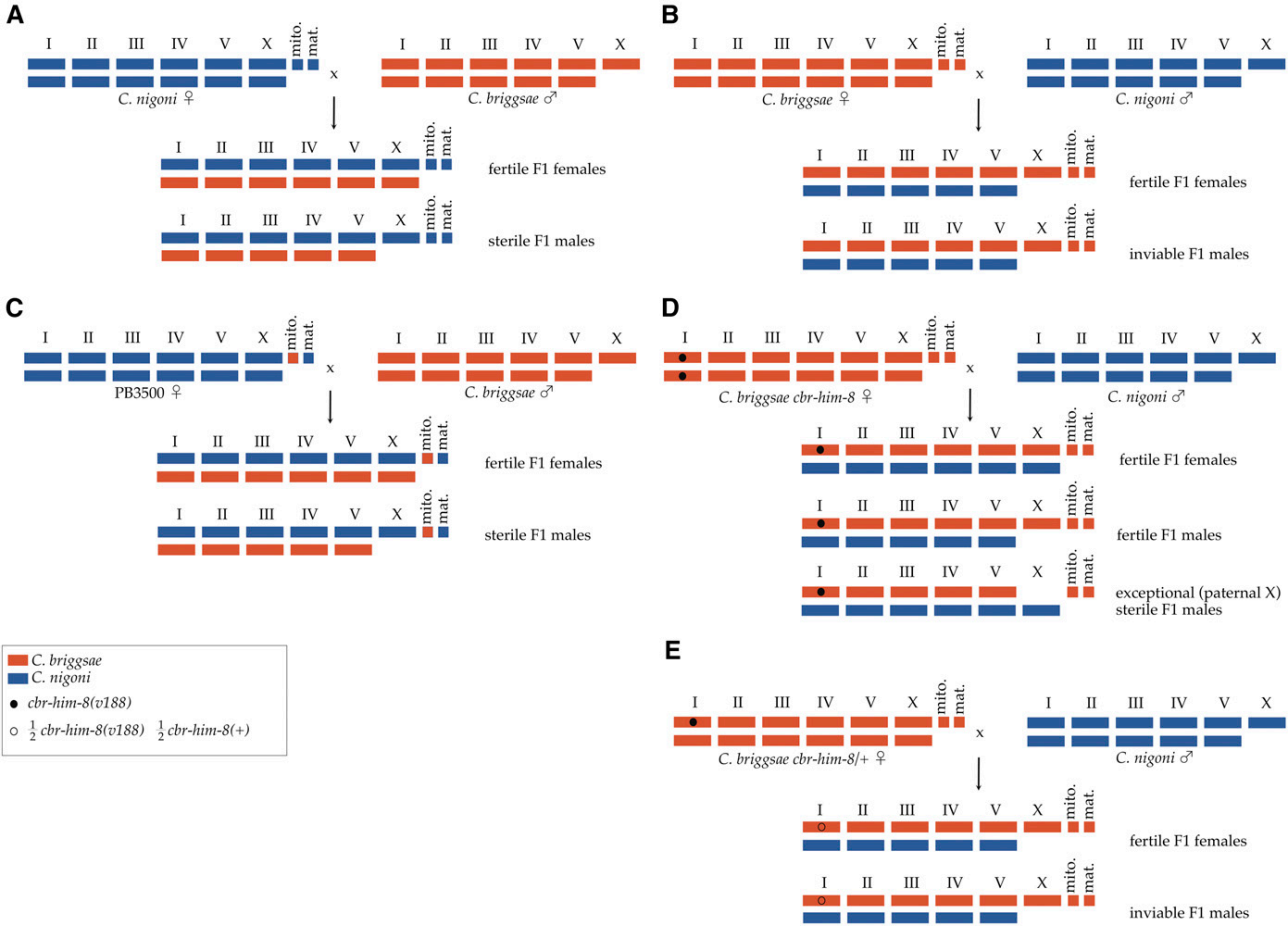
Chromosome and mitochondrial segregation and maternal contributions in *C. briggsae* × *C. nigoni* hybrid crosses. In all panels, *C. briggsae* and *C. nigoni* genotypes are indicated in red and blue, respectively. In F1 hybrids, maternal chromosomes are shown above paternal chromosomes. In panels D and E, the *v188* mutant allele of *cbr-him-8* is indicated by a closed circle on chromosome I. In panel E, an open circle on chromosome I indicates that half of F1 hybrids were expected to be heterozygous for *cbr-him-8(v188)*. Diagrammed are crosses between (A) *C. nigoni* females and *C. briggsae* males, (B) sperm-depleted *C. briggsae* hermaphrodites and *C. nigoni* males, (C) PB3500 cybrid females and *C. briggsae* males, (D) sperm-depleted *C. briggsae cbr-him-8* mutant hermaphrodites and *C. nigoni* males, and (E) sperm-depleted *C. briggsae cbr-him-8/+* heterozygous hermaphrodites and *C. nigoni* males.

Asymmetric results were observed in reciprocal crosses between the *Caenorhabditis* species *C. nigoni* and *C. briggsae* (Figure 2, A and B, and Table 1). Despite considerable embryonic lethality, viable and fertile F1 hybrid females were obtained from both cross directions. From *C. nigoni* mothers, some viable but sterile F1 hybrid males were obtained. However, from *C. briggsae* mothers, all F1 hybrid males died during embryogenesis. These results were consistent with results reported by Woodruff *et al.* (2010) and Kozlowska *et al.* (2011). Woodruff *et al.* (2010) reported no viable males from 186 F1s scored. Kozlowska *et al.* (2011) reported only seven viable males from 3705 F1s scored. Similarly, from 429 F1s scored in this study, no viable F1 males were observed (Table 1).

**Table 1:** Frequency of F1 males derived from *C. briggsae* mothers

Cross	♀♀	♂♂	Fract. ♂	♂ Fract. X^Cbr^ (N)
*C. briggsae* AF16 ♂♂ × *C. nigoni* EG5268 ♀♀*^a^*	293	32	0.098*^b^*	
*C. nigoni* EG5268 ♂♂ × *C. briggsae* AF16 ♀♀*^c^*	429	0	0.000	
*C. briggsae* AF16 ♂♂ × PB3500 cybrid ♀♀*^d^*	383	39	0.092*^b^*	
*C. nigoni* EG5268 ♂♂ × *C. briggsae* RE980 ♀♀*^e^*	330	68	0.171	
*C. nigoni* EG5268 ♂♂ × *C. briggsae* PB192 ♀♀*^e^*	634	142	0.183	0.60 (131)*^f^*
*C. nigoni* EG5268 ♂♂ × *C. briggsae cbr-him-8(v188)* ♀♀*^e^*^,^*^g^*	964	210	0.179	

AF16, *C. briggsae* wild-isolate; EG5268, *C. nigoni* wild-isolate; PB3500, EG5268 nuclear genome and AF16 mitochondria; RE980, *C. briggsae* cbr-him-8(v188) I; PB192, *C. briggsae* cbr-him-8(v188) I; stIs20120 [pmyo2::GFP] X (RE980 and PB192 are both AF16 derivatives).

a,c,d,e These crosses are diagrammed in Figure 2, A, B, C, and D, respectively.

b ♂ frequencies not significantly different, *P* = 0.677 chi squared test, expected frequency = 0.098.

f Sum of results from crosses using RE980 and PB192 ♀♀.

g Pharyngeal expression of GFP observed in 79 of 131 F1 males scored.

F1 males from reciprocal crosses in *Caenorhabditis* differed in the source of their maternally derived X chromosome, their maternally derived mitochondria, and in maternal contributions to the oocyte prior to fertilization (Figure 2). These differences have been proposed as potential causes of asymmetric results in reciprocal crosses (Turelli and Moyle 2007; Dey *et al.* 2014). To test for dysgenic mitonuclear interactions, *C. briggsae* males were mated to females from the PB3500 cybrid strain. This strain possessed a *C. nigoni* nuclear genome and *C. briggsae* mitochondria (Figure 1). Viable F1 males were obtained from this cross. Frequencies of F1 males obtained from crosses of *C. briggsae* AF16 males to *C. nigoni* EG5268 and cybrid PB3500 mothers were identical (Figure 2C and Table 1). As mitochondria are maternally inherited, males derived from PB3500 mothers would have possessed *C. briggsae* mitochondria. The viability of these F1 males is not consistent with dysgenic mitonuclear interactions as a cause of F1 male-specific lethality of F1 males derived from *C. briggsae* mothers. This result was consistent with those of Bundus *et al.* (2015), who found that *C. briggsae* mitochondria did not have an impact on postzygotic reproductive isolation in crosses between *C. briggsae* and *C. nigoni*.

To discriminate between maternal-zygotic and X-autosomal interactions, *C. nigoni* males were mated to sperm-depleted *C. briggsae cbr-him-8(v188) I* hermaphrodites. The *cbr-him-8(v188)* mutation results in high rates of X chromosome nondisjunction and hence in high frequencies of XO males among self-progeny of mutant hermaphrodites (Wei *et al.* 2013). It was thought that this cross would produce exceptional males with a paternal *C. nigoni* X chromosome (X^Cni^) through the fertilization of nullo-X oocytes by X-bearing sperm. Viability of these males would eliminate *C. briggsae* maternal-zygotic interactions as the cause of asymmetric F1 male-specific lethality. Viable F1 males were obtained from *C. briggsae cbr-him-8(v188)* mutant mothers (Figure 2D and Table 1). However, only 40% of these were the expected exceptional X^Cni^ males (Table 1). The rest of the viable F1 males possessed a maternally derived *C. briggsae* X (X^Cbr^) chromosome. This was determined from crosses of *C. nigoni* males to hermaphrodites from the PB192 strain of *C. briggsae*. PB192 is an AF16 derivative that was mutant for *cbr-him-8(v188)* and that also included an X-linked insertion, *stIs20120*, of a *cbr-myo2p*::*GFP* transgene. Expression from *stIs20120* results in pharyngeal GFP fluorescence (Yan *et al.* 2012). Frequencies of F1 males obtained from crosses that included or did not include *stIs20120* were identical (Table 1). As PB192 is an AF16 derivative, the only difference between the viable X^Cbr^ F1 males derived from PB192 mothers and the inviable X^Cbr^ F1 males derived from wild-type AF16 *C. briggsae* mothers was the presence of *cbr-him-8(v188)* and *stIs20120*. In control crosses, *stIs20120* was shown to have no effect on F1 male viability (not shown). For viable X^Cbr^ F1 males, *cbr-him-8* was homozygous in the maternal genome and heterozygous in the zygotic genome. Hence, *cbr-him-8(v188)* was identified as a suppressor of the lethality of F1 X^Cbr^ males.

### Suppression of hybrid by cbr-him-8(v188) is a maternal effect

In *Caenorhabditis elegans*, mutations in *him-8* exhibit two distinct and separable phenotypes. Homozygosity of *him-8* results in high rates of X chromosome nondisjunction (Hodgkin *et al.* 1979). This is caused by defects in X chromosome pairing during meiosis (Phillips *et al.* 2005). In somatic cells, *him-8* mutations are dominant suppressors of missense mutations in transcription factor binding domains (Nelms and Hanna-Rose 2006; Sun *et al.* 2007). If *C. briggsae cbr-him-8(v188)* exhibits both of these phenotypes, then suppression of F1 male-specific lethality could be the result of maternal homozygosity or zygotic heterozygosity.

To distinguish between maternal and zygotic modes of suppression, *C. nigoni* males were crossed with *cbr-him-8/+ C. briggsae* heterozygotes. The X chromosome nondisjunction phenotype of *cbr-him-8(v188)* is recessive. If suppression results from X chromosome pairing defects during meiosis, then few if any F1 males would be expected from *cbr-him-8* heterozygous mothers. Conversely, half of F1 male progeny from heterozygous mothers would inherit the mutant allele of *cbr-him-8*. These males would be genetically identical to F1 males derived from *cbr-him-8* homozygotes. If suppression results from somatic suppression of transcription factor binding defects, then the abundance of viable F1 X^Cbr^ males derived from heterozygous mothers would be expected to be half of that observed from *cbr-him-8* homozygotes. From crosses of *C. nigoni* males to *C. briggsae cbr-him-8/+* hermaphrodites, a single F1 male was observed among 354 viable F1 progeny scored (Figure 2E and Table 2). This result excludes zygotic suppression but is consistent with maternal pairing defects as the cause of suppression of X^Cbr^ F1 male-specific lethality.

**Table 2:** Tests of zygotic and maternal suppression hypotheses

Observed	Females	Males	*P* value*^a^*
*C. nigoni* × *C. briggsae cbr-him-8/+**^b^**^,^**^c^*	353	1	
Expected			
Zygotic suppression*^d^*	331.3	22.7	2.567 × 10^−6^
Maternal suppression*^e^*	353.3	0.7	0.685

a *P* values from chi squared tests using the expected male frequencies for the zygotic and maternal suppression hypotheses described above.

b *C. nigoni* EG5268 ♂♂ × *C. briggsae* cbr-him-8(v188)/+ I; stIs20120 [p-myo2::GFP] X, or *C. nigoni* EG5268 ♂♂ × *C. briggsae* cbr-him-8(v188)/+ I; stIs20120 [p-myo2::GFP]/+ X.

c This cross is diagrammed in Figure 2E.

d An expected male frequency of 6.4% was based on the expected 50% transmission rate of *cbr-him-8(v188)* from maternal heterozygotes and on the 12.8% frequency of viable adult X^C^b^r^ males from *cbr-him-8(v188)* homozygous mothers.

e An expected male frequency of 0.19% was based on the frequency of viable males obtained from crosses of *C. nigoni* males to wild-type *C. briggsae* hermaphrodites (Kozlowska *et al.* 2011).

### F1 X^Cbr^ males are fertile

**Figure 3:**
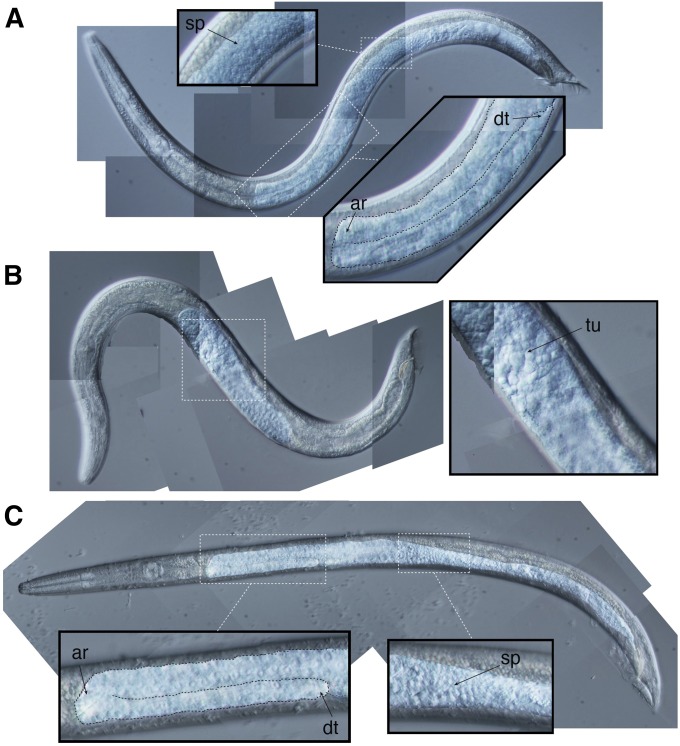
Gonad morphology in F1 male hybrids. (A) *C. nigoni* EG5268, (B) F1 X^Cni^, and (C) F1 X^Cbr^ males. Contrast of gonads enhanced in all panels. Boxes correspond to regions enlarged in insets. In panels A and C, the distal arm is outlined with a dashed line in the large insets to emphasize the tubular structure of the gonad. This tubular structure is absent in the F1 X^Cni^ male shown in panel C. Anterior reflex (ar), distal tip (dt), sperm (sp), and tumorous cells (tu) indicated in insets. The *C*. F1 X^Cni^ male was an ‘exceptional’ GFP^–^ male obtained from crosses on *C. nigoni* EG5268 males to *C. briggsae* PB192 [*cbr-him-8(v188) I*; *stIs20120 (pmyo2*::*GFP) X*] hermaphrodites. The F1 X^Cbr^ male was a GFP^+^ male obtained from the same cross.

F1 X^Cbr^ males derived from crosses of *C. nigoni* males to *C. briggsae cbr-him-8* mutant hermaphrodites had well-developed gonads and were fertile (Figure 3 and Table 3). When F1 X^Cbr^ males were crossed to *C. nigoni* females, fertilized embryos were observed. All of these embryos arrested prior to hatching. When F1 X^Cbr^ males were mated to *C. briggsae* hermaphrodites, viable F2 adult progeny were obtained. When F1 X^Cbr^ males were crossed to F1 females, the result varied depending upon the source of F1 females. When crossed to F1 females derived from *C. nigoni* mothers (F1^Cni^ females), only arrested embryos were observed. When crossed to F1 females derived from *C. briggsae* mothers (F1^Cbr^ females), viable F2 adults were obtained approximately a third of the time. Further crosses will be required to determine if these differences are significant.

**Table 3:** Fertility of F1 X^C^b^r^ males

Cross*^a^*	Result*^b^*	Self-Fertile F2	F2 Male
		Female Fraction N*^c^*	Fraction (N)
F1 X^Cbr^ ♂ × *C. nigoni* ♀	Dead embryos (5)		
	No progeny (3)		
F1 X^Cbr^ ♂ × *C. briggsae* ♀	Viable adults (16)	0.98 (48)	0.20 (869)
F1 X^Cbr^ ♂ × F1^Cni^ ♀	Dead embryos (2)		
F1 X^Cbr^ ♂ × F1^Cbr^ ♀*^d^*	Dead embryos (6)		
	Viable adults (3)	nd*^f^*	∼0.50*^e^*
	Viable adults (1)	1.00 (30)	0.005 (208)
	No progeny (1)		

a F1 X^C^b^r^ ♂ = GFP^+^ males derived from PB192 mothers, F1 ♀^Cni^ = F1 females derived from *C. nigoni* mothers. F1 ♀^Cbr^ = F1 females derived from *C. briggsae* mothers.

b Number of crosses for each given result indicated in parentheses.

c Fraction of anatomically female (*i.e.*, XX) F2s that laid eggs. Number scored indicated in parentheses.

d Includes results of full sib crosses as well as results of F1 X^Cbr^ males from PB192 mothers crossed to F1 females from AF16 mothers.

e F2 males abundant but not counted. It is not clear why males were abundant in some crosses but not in others.

f Not done.

Adult male, female and hermaphrodite progeny were obtained from crosses of F1 X^Cbr^ males to *C. briggsae* hermaphrodites and F1^Cbr^ females (Table 3). However, the frequencies of these different progeny types were not consistent with expectations. Among cross progeny, haplo-X males were expected at a frequency of 0.50. From crosses to *C. briggsae* hermaphrodites, observed frequency of males, 0.20, was significantly lower than this expectation (*P* < 0.0001). From crosses to F1^Cbr^ females, F2 males were sometimes, but not always, abundant. From both crosses, nearly all diplo-X progeny were self-fertile. Self-sterile (female) and self-fertile (hermaphrodite) diplo-X progeny were both expected from these crosses. However, Woodruff *et al.* (2010) demonstrated that self-sterility (female reproductive mode) was dominant and they observed very low frequencies (< 3%) of self-fertility among progeny of 2^nd^ or 3^rd^ generation hybrid males crossed to *C. briggsae* hermaphrodites. The high rates of self-fertility, ≥ 0.98, observed among diplo-X progeny was not consistent with this observation.

### F1 X^Cni^ males are sterile regardless of cross direction

F1 X^Cni^ males derived from *C. nigoni* mothers have gonad defects and are sterile (Woodruff *et al.* 2010). In general, these F1 males were defective in gonadal outgrowth (Table 4). Gonad outgrowth in *C. nigoni* and *C. briggsae* is nearly identical to gonad outgrowth in *C. elegans*. Gonad outgrowth in *C. elegans* is regulated by the migration of the linker cell (Kimble and Hirsh 1979; Kato and Sternberg 2009). The linker cell initially migrates anteriorly along the ventral body wall until the L2 larval molt. It then migrates to the dorsal body wall where it turns and migrates posteriorly during the L3 and L4 larval stages. The result of these migrations is a thin tubular gonad with an anterior reflex (180° bend) near the posterior bulb of the pharynx. In some F1 X^Cni^ males, there is an apparent complete failure in gonad outgrowth. These males possess gonads that differ little from the gonad primordium present in L1 larvae at hatching. In other F1 X^Cni^ males, there is an apparent failure in the dorsal turn of the linker cell at the L2 molt. These males possess swollen, ovoid gonads that lack an anterior reflex (Table 4).

**Table 4:** Gonadal phenotypes of F1 X^Cni^ males

Cross	No Outgrowth*^a^*	Defective Outgrowth*^b^*	N
*C. briggsae* AF16 ♂♂ × *C. nigoni* EG5268 ♀♀	9	10	19
*C. briggsae* PB192 ♂♂ × *C. nigoni* EG5268 ♀♀	6	15	21*^c^*
*C. nigoni* EG5268 ♂♂ × *C. briggsae* PB192 ♀♀	5	2	7*^c^*

AF16, *C. briggsae* wild-isolate; EG5268, *C. nigoni* wild-isolate; PB192, *C. briggsae* cbr-him-8(v188) I.

a Small ventral ovoidal masses of gonadal tissue, or degenerate vacuoles, located at midbody.

b Larger masses of gonadal tissue extending anteriorly toward the pharynx but lacking the anterior reflex. Differentiated and/or tumorous cells often observed.

c Distributions of gonadal phenotypes in X^Cni^ males derived from PB192 ♂♂ × EG5268 ♀♀ and EG5268 ♂♂ × PB192 ♀♀ do not differ significantly from the distribution of phenotypes derived from AF16 ♂♂ × EG5268 ♀♀. *P* values 0.084 and 0.20, respectively.

F1 X^Cni^ males obtained from *C. briggsae cbr-him-8(v188)* mutant mothers had the same gonadal outgrowth defects as those observed in F1 X^Cni^ males derived from *C. nigoni* mothers (Figure 3 and Table 4). The only genetic difference between these males and their F1 X^Cbr^ male siblings, which had well-developed functional gonads, was the X chromosome. Based on these results, the gonadal outgrowth defects observed in F1 X^Cni^ males must result from the presence of a hybrid sterile gene on the X chromosome of *C. nigoni*.

## Discussion

In crosses between *C. nigoni* males and *C. briggsae* hermaphrodites, almost all F1 male hybrids die during embryogenesis. This F1 hybrid male-specific lethality was suppressed by the *cbr-him-8(v188)* mutation. This result was unexpected. There is evidence that F1 male-specific lethality results from dysgenic interactions between a *C. briggsae* X-linked locus and *C. nigoni* autosomal loci (Bi *et al.* 2015). *cbr-him-8* does not correspond to this X-linked gene as it is located on chromosome I (www.wormbase.org). Rather, *cbr-him-8* must be acting as a suppressor of this hybrid lethality gene.

In *C. elegans*, mutations in *him-8* are pleiotropic. The HIM-8 protein binds to the pairing centers of the X chromosomes and is required for the meiotic pairing of X chromosomes (Phillips *et al.* 2005). Consequences of disrupted meiotic pairing include X-specific nondisjunction and an expansion of recombination distances on the X chromosome (Hodgkin *et al.* 1979; Broverman and Meneely 1994). The X-specific nondisjunction phenotype of *cbr-him-8(v188)* and the conservation of HIM-8 proteins in these species indicate that the role of HIM-8 in X chromosome pairing is conserved in *C. briggsae* (Phillips and Dernberg 2006; Wei *et al.* 2013). *C. elegans him-8* mutations also act as dominant suppressors of missense mutations in the DNA-binding domains of transcription factors (Nelms and Hanna-Rose 2006; Sun *et al.* 2007). Conservation of this phenotype in *C. briggsae* has not been tested.

The suppression of F1 male-specific lethality by *cbr-him-8* likely results from defects in X chromosome meiotic pairing during oogenesis in *C. briggsae*. This was evident from crosses of *C. nigoni* males to *C. briggsae cbr-him-8*/+ hermaphrodites. The X-nondisjunction phenotype, and hence the pairing defects, of *cbr-him-8* are recessive. However, half the F1 hybrids derived from *cbr-him-8*/+ heterozygous mothers would also have been heterozygous for *cbr-him-8*. Thus, the absence of viable F1 male progeny from *cbr-him-8*/+ mothers demonstrates that zygotic heterozygosity of *cbr-him-8(v188)* is not sufficient to suppress male-specific lethality in F1 hybrids.

The suppression of F1 male-specific lethality by *cbr-him-8* may result from meiotic silencing of the *C. briggsae* X chromosomes during oogenesis or from epigenetic suppression of X-linked gene expression during embryogenesis. In *C. elegans*, unpaired chromosomes are dimethylated on lysine 9 of histone H3 (H3K9me2) during meiosis (Bean *et al.* 2004; Bessler *et al.* 2010). H3K9me2 is a highly conserved epigenetic mark that is associated with transcriptional repression and meiotic silencing (Turner 2007; Kelly and Aramayo 2007; Kota and Feil 2010; Maine 2010; Mozzetta *et al.* 2015). Acquisition of H3K9me2 on unpaired X chromosomes in *C. elegans her*-1 XO hermaphrodites is associated with meiotic repression of transcription of X-linked genes (Bean *et al.* 2004). However, the repressive epigenetic imprint acquired by the X chromosome during spermatogenesis can also persist through the 14-cell stage of embryogenesis (Kelly *et al.* 2002; Bean *et al.* 2004). It should be possible to test for suppression by meiotic silencing by generating a mutation in *cbr-met-2*. In *C. elegans*, *met-2* is required for dimethylation of H3K9 (Bessler *et al.* 2010). If suppression of F1 male-specific lethality results from H3K9me2 of X chromosomes in *cbr-him-8* mutant hermaphrodites, then X^Cbr^ F1 males derived from *cbr-him-8*; *cbr-met-2* doubly mutant hermaphrodites should die during embryogenesis.

Our results also demonstrated that the sterility of X^Cni^ F1 males was caused by dysgenic interactions between the X chromosome of *C. nigoni* and the autosomes of *C. briggsae*. From *C. briggsae cbr-him-8* mothers, both X^Cni^ and X^Cbr^ F1 males were obtained. The X^Cbr^ F1 males had well-developed gonads and were fertile whereas their X^Cni^ siblings had defects in gonad development and were sterile. The X^Cbr^ and X^Cni^ males obtained from these crosses shared the same maternal and mitochondrial genotypes. They differed only in the identity of their X chromosomes. Moreover, unpaired X chromosomes in male spermatogenesis (*i.e.*, X^Cni^) were expected to share similar epigenetic modifications as unpaired X chromosomes in hermaphrodite oogenesis in *cbr-him-8* mutant mothers (Bean *et al.* 2004). Thus, the cryptic asymmetry observed in F1 male fertility likely results from the divergence of one or more loci on the *C. briggsae* and *C. nigoni* X chromosomes.

Finally, the fertility of F1 X^Cbr^ males provides an opportunity to define the genetic requirements for hermaphroditic reproduction in *C. briggsae*. Woodruff *et al.* (2010) demonstrated that the hermaphroditic mode of reproduction was recessive to the female mode in diplo-X hybrids. We found females to be rare among diplo-X backcross progeny of X^Cbr^ males mated to *C. briggsae* hermaphrodites. Genotyping of these rare backcross females should allow for the identification of *C. nigoni* loci that suppress spermatogenesis in female hybrids.
